# An Unusual Presentation of Inferior Mesenteric Artery Steal Syndrome Induced by Walking

**DOI:** 10.7759/cureus.87127

**Published:** 2025-07-01

**Authors:** Affaf Ahmed, Mohammed H Abdelaty, Lorraine Corfield

**Affiliations:** 1 Department of Vascular Surgery, University Hospitals of North Midlands NHS Trust, Stoke-on-Trent, GBR

**Keywords:** aortobifemoral bypass, aortoiliac occlusive disease, inferior mesenteric artery steal, mesenteric ischemia, mesenteric steal syndrome, surgical case report

## Abstract

Aortoiliac occlusive disease results in varying degrees of pelvic and lower extremity arterial insufficiency. Disease limited to the infrarenal segment does not typically affect intestinal perfusion in the absence of visceral aortic or mesenteric vessel involvement. We report a rare case of a 77-year-old woman who presented with severe and incapacitating abdominal pain triggered by walking short distances (approximately 20 yards). Computed tomography angiography demonstrated that the coeliac and superior mesenteric arteries were patent, but the infrarenal aorta and both common iliac arteries were occluded. Collaterals from the inferior mesenteric artery (IMA) supplied the lower limbs. The abdominal pain was thought to be due to the arterial supply to her legs from the diverting mesenteric blood flow to supply the lower limbs, resulting in mesenteric ischemia when walking. The patient underwent a successful aortobifemoral bypass, which resulted in the resolution of her abdominal symptoms. IMA steal has rarely been reported in the literature, and vascular surgeons should be aware of this unusual differential diagnosis for "abdominal pain on walking."

## Introduction

Aortoiliac occlusive disease (AIOD) is one form of peripheral arterial disease (PAD) that usually involves the infrarenal abdominal aorta and the iliac arteries. Like other types of PAD, atherosclerosis is the main aetiology, and common risk factors include smoking, diabetes, hypertension, and hyperlipidaemia [1]. Patients can remain asymptomatic for a long duration depending on the extent of arterial collateralization, particularly between branches of the inferior mesenteric, internal iliac, and profunda femoris arteries [2]. Common symptoms include intermittent claudication, ischemic rest pain, and tissue loss. The latter two symptoms are usually referred to as critical limb ischemia. Another form of PAD is mesenteric vascular disease, in which one or more of the mesenteric arteries are affected by atherosclerosis. This can result in a condition known as chronic mesenteric ischemia. Those patients usually present with long-standing post-prandial abdominal pain, food fear, and weight loss [3]. In this article, we present an unusual case of abdominal pain with walking, which was caused by AIOD in the absence of mesenteric arterial lesion.

## Case presentation

History

A 77-year-old woman presented with severe and incapacitating abdominal pain on walking approximately 20 yards around her home. The pain settled within five minutes after rest. She did not have this abdominal pain at any other time. Past medical history included rheumatoid arthritis for which she took methotrexate, as well as hypertension. Also, she was a recent ex-smoker. One year previously, she was diagnosed with cervical and lumbosacral nerve root compression.

Clinical examination

Abdominal examination was unremarkable. She had no palpable femoral pulses bilaterally, but her feet were warm and well perfused with a negative Buerger’s test. Her straight leg raise was negative, though she did have some pain on right hip abduction.

Investigations

Blood tests showed no significant abnormalities (Table 1).

**Table 1:** Laboratory values of the patient. INR: international normalized ratio; aPTT: activated partial thromboplastin time; eGFR: estimated glomerular filtration rate

Components	Value	Units	Range
Haemoglobin	114	g/L	115-165
White cell count	10.65	10^9^/L	4.0-11.0
Platelets	427	10^9^/L	150-450
Red blood cell count	3.43	10^12^/L	3.80-5.80
Haematocrit	0.333	L/L	0.36-0.47
Mean cell volume	97.0	fL	80-100
Mean cell haemoglobin	33.3	pg	27-32
Neutrophils	9.25	10^9^/L	2.0-7.5
Lymphocytes	0.78	10^9^/L	1.5-4.0
Monocytes	0.50	10^9^/L	0.20-0.80
Eosinophils	0.07	10^9^/L	0.04-0.40
Basophils	0.05	10^9^/L	0.00-0.10
Nucleated red blood cells	<0.2	10^9^/L	-
C-reactive protein	4	mg/L	0-5
INR	1.0		0.8-1.2
aPTT ratio	0.94		0.8-1.17
Ferritin	49	µg/L	12-240
Albumin	35	g/L	35-50
Total bilirubin	5	µmol/L	0-21
Alkaline phosphate	83	IU/L	30-130
Alanine transaminase (ALT)	14	IU/L	0-34
Sodium	134	mmol/L	133-146
Potassium	4.3	mmol/L	3.5-5.3
Urea	4.9	mmol/L	2.5-7.8
Creatinine	68	µmol/L	45-84
eGFR result (EPI)	74	ml/min/1.73m^2^	-
Thyroid-stimulating hormone	3.64	mIU/L	0.38-5.33

**Figure 1:**
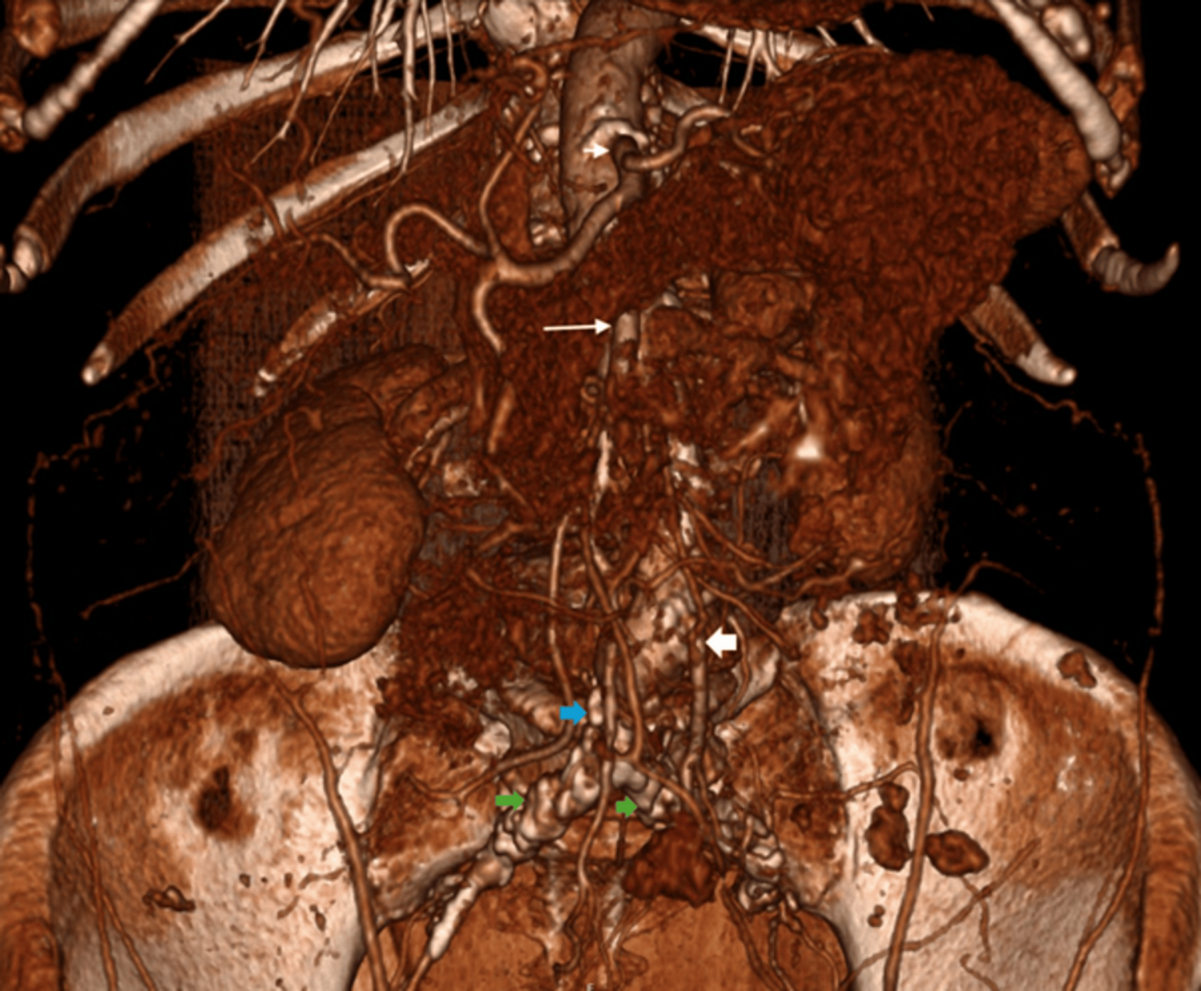
3D reconstruction of CTA showing a patent coeliac axis (short white arrow), patent superior mesenteric artery (long white arrow), and patent inferior mesenteric artery (thick white arrow). Also noted are an occluded distal aorta (blue arrow) and bilateral common iliac arteries (green arrows). CTA: computed tomography angiography

**Figure 2:**
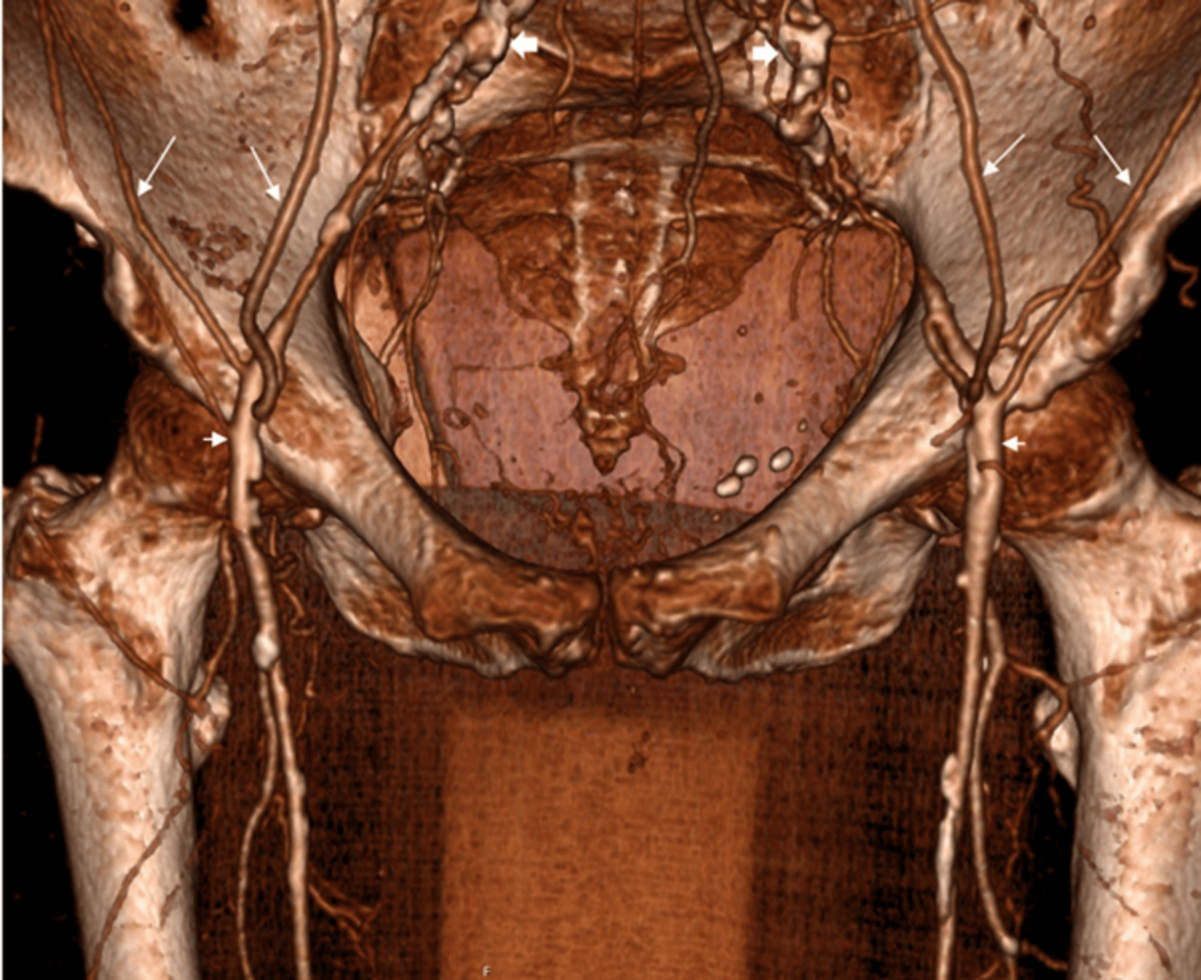
3D reconstruction of CTA showing occluded common iliac arteries (thick white arrows), bilateral patent common femoral arteries (short white arrows), which are supplied by collateral vessels (long white arrows). CTA: computed tomography angiography

**Figure 3:**
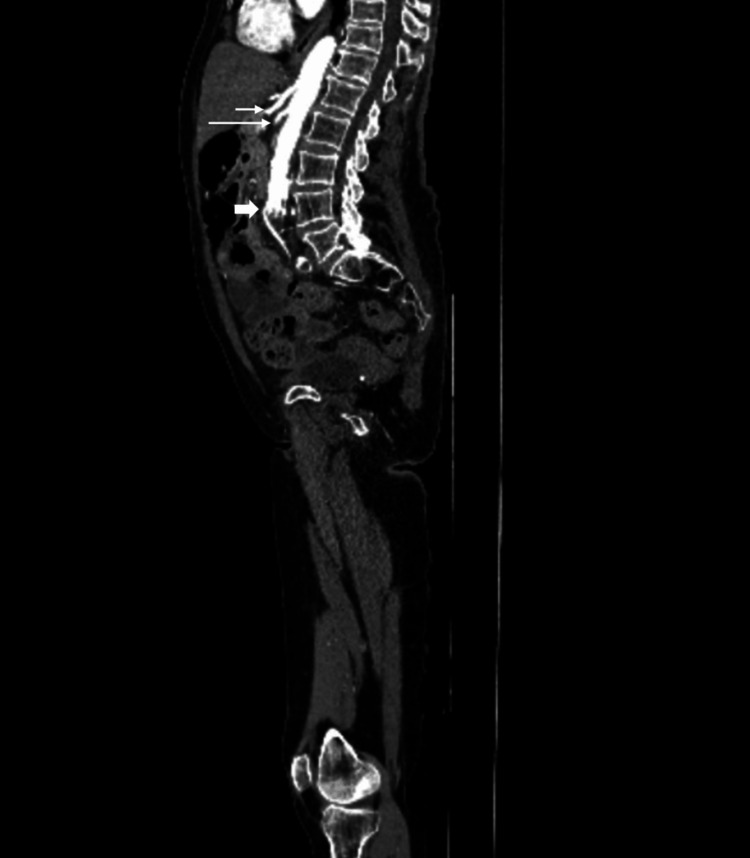
A sagittal section of computed tomography angiography (CTA) showing a patent origin of the coeliac trunk (short white arrow), superior mesenteric artery (long white arrow), and inferior mesenteric artery (thick white arrow).

The CT angiogram showed a patent coeliac, superior mesenteric artery (SMA), and inferior mesenteric artery (IMA). Distal aortic and common iliac arteries were occluded with IMA collaterals feeding her leg vessels (Figures 1-3).

Treatment

Her abdominal pain was attributed to IMA steal secondary to the aortoiliac occlusion. This phenomenon represents a mesenteric steal, where exercise-induced increased demand from the lower limbs diverts blood flow away from the mesenteric circulation, resulting in transient intestinal ischemia. The decision was made to proceed with aortobifemoral bypass. She was commenced on 75mg of clopidogrel and 80mg of atorvastatin as part of the best medical treatment for PAD.

A preoperative workup was done to assess surgical fitness for aortobifemoral bypass, which included a myocardial perfusion (MIBI) scan showing normal ejection fraction and no inducible ischemia. Pulmonary function tests showed normal lung volumes and mild airway obstruction. The operation went smoothly, and she had a good post-operative recovery apart from an uncomplicated urinary tract infection. She was seen in the clinic about seven weeks post-surgery, and her symptoms had resolved.

## Discussion

AIOD typically begins at the aortic terminus and common iliac artery origins and slowly progresses proximally and distally. Progression is variable but may ultimately result in total aortic occlusion. When collaterals are adequate, claudication symptoms are often tolerable and can be successfully managed nonoperatively for many years. In our patient, despite patency of the mesenteric arteries, the high resistance due to distal aortoiliac occlusion likely diverted flow through collateral pathways during ambulation, effectively "stealing" mesenteric perfusion and resulting in transient gut ischemia. Historically, the surgical options for AIOD included aortoiliac endarterectomy, aortoiliac bypass, aortobifemoral bypass, and extra-anatomic bypass (iliofemoral, femorofemoral, or axillofemoral). Given the superior long-term patency, aortobifemoral grafting is currently considered the open revascularization procedure of choice unless the patient is a poor surgical candidate [4].

Endovascular techniques have also shown reasonable outcomes. Recent trials have demonstrated promising results for covered endovascular reconstruction of the aortic bifurcation (CERAB) in terms of patency. A recent study by Kruszyna et al. showed one-year primary patency of 94.5% [5]. Another study by Rouwenhorst et al. investigated the long-term results of CERAB, which showed a primary patency rate of 77.5% after five years [6].

IMA steal has been reported in the literature only a couple of times, to the best of our knowledge. A similar case described a patient having post-prandial abdominal pain that was also provoked by walking. In this case, the mesenteric arteries were diseased, so treatment was directed to recanalize them. Endovascular treatment was attempted but followed by recurrence. The patient then underwent surgical transposition of the IMA to the left common iliac artery, which significantly improved the symptoms. In our case, the occlusion affected mainly the aortoiliac segment, while the mesenteric vessels remained patent. Therefore, aortobifemoral bypass was done and resulted in the resolution of the symptoms [7].

Another case reported by Mirza and Bacharach had chronic mesenteric ischemia manifestations along with lower limb claudication. Similar to our patient, the main pathology was in the infrarenal aorta. However, a short-segment stenosis was found in their patient. Therefore, the authors preferred endovascular treatment with angioplasty and an aortic stent. In contrast, our patient had extensive aortoiliac occlusion. Therefore, we opted for surgical revascularization for higher success rates and long-term patency [8].

This case is unique in having abdominal pain with walking despite not having any mesenteric arterial disease, and to our knowledge, it differs from previously reported cases.

## Conclusions

This particular case is a reminder of the unusual presentation of abdominal pain with walking, which can be due to mesenteric steal in the setting of aortoiliac disease.

Although rare, IMA steal should be ruled out in patients presenting with abdominal pain on walking. The rich collateral circulation between the IMA and lower limb arteries can result in re-direction of the blood from the gut to the lower limb during walking, resulting in chronic mesenteric ischemia symptoms. Surgical treatment of the occluded aortoiliac segment can result in significant improvement.
